# Ectopic Neo-Formed Intracellular Membranes in *Escherichia coli*: A Response to Membrane Protein-Induced Stress Involving Membrane Curvature and Domains

**DOI:** 10.3390/biom8030088

**Published:** 2018-09-04

**Authors:** Nadège Jamin, Manuel Garrigos, Christine Jaxel, Annie Frelet-Barrand, Stéphane Orlowski

**Affiliations:** 1 Institute for Integrative Biology of the Cell (I2BC), CEA/Institut des Sciences du Vivant Fréderic-Joliot/SB2SM, CNRS UMR 9198, Université Paris-Sud, Université Paris-Saclay, 91191 Gif sur Yvette CEDEX, France; manuel.garrigos@cea.fr (M.G.); christine.jaxel@cea.fr (C.J.); 2 Institut FEMTO-ST, UMR CNRS 6174, Université Bourgogne Franche-Comté, 15B avenue des Montboucons, 25030 Besançon CEDEX, France; annie.frelet-barrand@femto-st.fr

**Keywords:** intracellular membranes, *Escherichia coli*, membrane domains, vesicles, tubules, membrane curvature, membrane protein overexpression

## Abstract

Bacterial cytoplasmic membrane stress induced by the overexpression of membrane proteins at high levels can lead to formation of ectopic intracellular membranes. In this review, we report the various observations of such membranes in *Escherichia coli*, compare their morphological and biochemical characterizations, and we analyze the underlying molecular processes leading to their formation. Actually, these membranes display either vesicular or tubular structures, are separated or connected to the cytoplasmic membrane, present mono- or polydispersed sizes and shapes, and possess ordered or disordered arrangements. Moreover, their composition differs from that of the cytoplasmic membrane, with high amounts of the overexpressed membrane protein and altered lipid-to-protein ratio and cardiolipin content. These data reveal the importance of membrane domains, based on local specific lipid–protein and protein–protein interactions, with both being crucial for local membrane curvature generation, and they highlight the strong influence of protein structure. Indeed, whether the cylindrically or spherically curvature-active proteins are actively curvogenic or passively curvophilic, the underlying molecular scenarios are different and can be correlated with the morphological features of the neo-formed internal membranes. Delineating these molecular mechanisms is highly desirable for a better understanding of protein–lipid interactions within membrane domains, and for optimization of high-level membrane protein production in *E. coli*.

## 1. Introduction: Membrane Domains in Bacteria

Biological membranes are pivotal in the life because they establish the structural frontier, defining a cell with respect to its environment, and hence forming a thermodynamic system. In eukaryotic cells, they also delimit the various intracellular organelles that are necessary for controlling the functional diversity of their metabolism. They are formed by a complex association between various lipids and membrane proteins, which account for about 30% of the genome, presenting an interrelated relationship. Indeed, if membrane proteins can be functionally modulated by the surrounding lipid molecules, some of which are also involved in lipid metabolism and trafficking. This functional diversity is coupled to a structural complexity, due to the transversal asymmetry and lateral heterogeneities of the lipid bilayer biomembranes. In particular, various membrane domains have been defined, including microdomains, nanodomains, lipid rafts, caveolae, lipid shells, membrane protein nanoclusters, and extensively addressed using various experimental approaches [1,2,3,4,5]. Such membrane domains are widely reported to be involved in various molecular and cellular processes [1,6,7,8,9], e.g., cellular lipid sorting and trafficking, functional regulation of membrane enzymes and transporters, lateral segregation of receptors forming signaling platforms, and entry portals for pathogenic agents. In particular, membrane domains are now well known to play a role in various cell membrane processes, including membrane turnover and remodeling, vesiculation, and cell shape alterations [1,10,11].

In contrast to eukaryotic cells, most bacteria are devoid of any internal membranes since they do not have any organelles (except purple photosynthetic bacteria, such as *Rhodobacter sphaeroides* [12]), and do not display internal membrane trafficking such as endocytosis. However, the bacterial cell membrane (single in Gram-positive or double in Gram-negative bacteria) is not that simple [13]. Indeed, it is now well known that the cytoplasmic membrane harbors regulated structures that are involved in important physiological functions [14,15,16,17]. In particular, specific curved regions, such as the bacterial poles and the septal ring, are described to be associated with specific enzymatic equipment [18,19]. In addition, diverse experimental approaches have demonstrated various bacterial membrane domains [20,21]. Such domains can be relevant at three levels. First, structurally, they are typically revealed by heterogeneous fluorescence staining [22,23,24] and by membrane protein subdiffusion compartments dependent on MreB polymerization [25]. Second, biochemically, they present specific lipid compositions such as cardiolipin (CL) [26,27,28], phosphatidylethanolamine (PE) [18,24], or hopanoids [29,30], specific proteins such as flotillins [20], and even resistance to detergents [31]. Finally, bacterial membrane domains can also be linked to specific functional characteristics, relying on multiprotein complexes such as transertion [14,32], MurG-dependent peptidoglycan synthesizing machinery [33], signaling transduction processes via the so-called “functional membrane microdomains” [16], oxidative phosphorylation complexes [34], and transient translocon complexes connecting inner-membrane proteins to outer-membrane clusters [35]. In all cases, the structural and functional characteristics of these bacterial membrane domains are interconnected.

## 2. The Bacterium *Escherichia coli* as an Unexpected Model for Addressing Molecular Mechanisms Underlying Membrane Morphology and Trafficking

The well-known Gram-negative *E. coli* is a bacterium that presents a high practical convenience for various aspects (culture media, generation time, protein mutations and engineering, protein overexpression, etc.), and is therefore widely used as a host system for the overexpression of a given membrane protein. Indeed, bacterial *E. coli* cells provide a more relevant biochemical environment in a living context compared to the pure, physico-chemically defined systems composed of artificial membranes, such as small, large, or giant unilamellar vesicles, or membranes extracted from cells such as giant plasma-membrane vesicles, which lack any metabolism. In terms of the biophysical features of membrane structure and function, *E. coli* provides a reliable biological context for studying some selected membrane proteins. In fact, this system enables the investigation of membrane protein function, within a specific membrane lipid composition in the case of a heterologous context, and protein structure after purification. However, heterologous overexpression of membrane proteins is often a difficult task because it may lead to toxicity for the host and/or unfolding and aggregation of the membrane protein within inclusion bodies. In few cases, overexpression (in particular at high levels) of a membrane protein in *E. coli* leads to ectopic neo-formed intracellular membranes. Since *E. coli* does not present internal membranes, this gives the opportunity to address this process within a relatively simplified context compared to eukaryotic cells, pointing out the involvement of membrane domains and organization in membrane morphology and trafficking. Hence, dissecting the molecular processes leading to internal neo-membrane formation should help to better control the production of high quality and quantity of membrane proteins production that is required for functional and structural studies. These structural studies may include the possible production of 2D crystals [36] as well as overloaded vesicles for cryo-electron microscopy [37] and solid state nuclear magnetic resonance (NMR) [38,39].

The scope of this review takes place within this frame, and is focused on observations made on *E. coli* membrane remodeling. Indeed, relatively rare events have been reported, which all reveal and enlighten the structuring roles of membrane domains, pointing out the importance of (specific or non-specific) membrane protein–lipid interactions as common underlying molecular mechanisms. These reports describe the effects of a membrane stress that is induced by the overexpression of membrane proteins at high levels, but also, more generally, by any mechanism of local overconcentration of a membrane protein. Here, we aim at collecting and reporting these data, and, for the first time to our knowledge, we classify them according to morphological characteristics. Furthermore, we address the molecular mechanisms underlying the formation and stabilization of these intracellular neo-membranes, and we finally propose a unified model based on the current concepts of membrane remodeling that are widely presented in other biological contexts, in order to delineate these mechanisms. These efforts will be useful from two points of view: (i) a basic perspective addressing some unusual aspects of the membrane and cellular consequences of interactions between lipids and membrane proteins, and (ii) an applied perspective considering the desired rational control and optimization of membrane protein overproduction in a bacterial host.

## 3. Membrane Stress Induced by Endogenous or Heterologous Membrane Protein Overexpression in *Escherichia coli*

The neo-formed, ectopic *E. coli* intracellular membranes described in the literature could be classified according to four morphological criteria: (i) tubules (presenting single curvature, i.e., cylindrically shaped with one main curvature plan) vs. vesicles (presenting double curvature, i.e., spherically shaped with two main curvature plans); (ii) stacked arrays (“ordered”) vs. entangled structures (“disordered”); (iii) mono- vs. poly-dispersion of size and shape; (iv) connected with vs. separated from the cytoplasmic membrane. The technique generally used to morphologically characterize these intracellular neo-membranes is electron microscopy, occasionally combined with fluorescent labelling experiments. These imaging techniques are most often completed by biochemical analyses of the proteo-lipid composition of these membranes. In the few cases where time-dependence of membrane neo-formation was reported, no qualitative evolution of the morphological characteristics of these membranes was observed.

### 3.1. Intracellular Tubules Connected to the Cytoplasmic Membrane (“Type I” Intracellular Neo-Membranes)

Five different overexpressions of *E. coli* membrane proteins have been reported to induce the formation of intracellular membrane tubules: the fumarate reductase complex (FrdABCD) [40], the *sn*-glycerol-3-phosphate acyl transferase (PlsB) [41], the fusion protein LamB-LacZ [42], the mannitol permease MtlA [43], and the chemotaxis receptor Tsr [44]. Large, 50–100-fold overproduction of MtlA led to both intracellular aggregates (likely inclusion bodies) and intracellular membrane tubular-shaped structures spread throughout the cytoplasm, which were not further characterized [43]. When the fusion protein of the outer membrane maltose porin with β-galactosidase (LamB-LacZ) was overexpressed in *E. coli*, a network of intracellular tortuous membrane tubules emanating from the cytoplasmic membrane was observed and strongly correlated to the level of protein expression; the overexpressed protein mainly had a cytoplasmic localization and was associated with these internal membranes [42].

The proliferating internal membranes generated by about 100-fold overexpression of Tsr formed tubular extensions with physical continuity from the cytoplasmic membrane, more or less extending deep inside the cell. These structures have branched and entangled morphologies (that could lead to apparent pseudo-vesicles), and are stabilized by interactions between the soluble, cytoplasmic, or periplasmic, domains of the protein. Moreover, the wild-type cells already presented few local invaginations at the poles where Tsr was endogenously expressed. This last observation illustrates Tsr influence on local membrane morphology when expressed at a physiological level. Thus, the formation of the neo-structures could be interpreted as resulting from the amplification of the physiological situation [44].

High overexpression rates of Frd and PlsB (20–40 and 35–50 fold, respectively) led to an overproduction by 50% of all the membrane proteins. This is concomitant with the appearance of an extra amount of internal membranes forming bundles of long tubules that are parallel to the cell axis underneath the cytoplasmic membrane (attached to it even after cell lysis), and containing the highly enriched protein-forming quasi-crystal 2D-ordered arrays [40,41,45,46]. For both protein overexpressions, the phospholipid-to-protein ratio was unchanged in the cytoplasmic membranes, but decreased within the tubule membranes when compared to the cytoplasmic membrane. For Frd overexpression, the phospholipids were enriched in CL and unsaturated fatty acid chains, although the CL enrichment in the tubules was less marked than in the cytoplasmic membrane [45]. Noteworthy, a mutant strain with a 5-fold decrease in CL content compared to wild-type cells overproduced Frd in membrane tubules, presenting altered morphologies and disorganized Frd protein 2D arrangement [45]. However, for PlsB, the lipid composition was globally unchanged [41] or, in another strain, CL percentage was increased [47]. Otherwise, and somehow unexpectedly, a heat shock response mutant strain did not show any intracellular tubule formation, but displayed the same levels of PlsB protein overexpression, without concomitant lipid metabolism modification, but with the presence of large inclusion bodies. Interestingly, in wild type cells, PlsB overexpression was shown to partially activate the heat shock response by inducing two chaperone proteins, GroEL and DnaK [47].

These data demonstrated the specificity of the protein–lipid composition of the tubule membranes compared to the cytoplasmic membranes, as well as the specificity of the biochemical features of each overexpressed membrane protein.

### 3.2. Intracellular Saccules/Cisternae and Multilamellar Structures (“Type II” Intracellular Neo-Membranes)

When the lipid A disaccharide synthase (LpxB) extrinsic membrane-associated protein from *E. coli* or *Haemophilus influenzae*, was about 300-fold overexpressed in *E. coli*, the protein production was accompanied by a 2-fold increase of phospholipid synthesis, including a 5-fold increase of CL proportion [48]. This stimulated metabolism was accompanied by the formation within the cells of membrane tubules of uniform diameter but various orientations, preferentially located at the cell periphery, but with no information about their connection to or separation from the cytoplasmic membrane.

Overexpression by 10–12 fold (producing about 20% of the total proteins) of the entire complex [49] or the b subunit [50,51] of the F_0_F_1_-ATP synthase of *E. coli* led to an array of intracellular cisternae-like and vesicular membrane structures that appeared beneath the cytoplasmic membrane, but were well separated from it. Noteworthy, such high protein overexpression led to bacterial cytotoxicity, which was not assessed for correlation to the energy metabolism. Indeed, bacterial growth was decreased and the remaining bacteria were elongated, whereas there were only minor effects on their survival, and the internal membrane formation for levels of protein expression was reduced to below 8% for total proteins. This suggests a saturating threshold of protein amount in the cytoplasmic membrane, leading to a bacterial stress and an adapted cell response [49]. A bacterial *E. coli* cell strain (C43) that is resistant to this stress has been selected, and it displayed much larger and denser tubular membrane networks than those of the initial strain (C41), while the classical BL21 strain produced no protein [50]. These tubular membranes were enriched to about 80% with the overexpressed protein [50]; this high density of protein allowed for interactions between adjacent membranes via the F_1_ domains [49]. The importance for internal tubules/cisternae formation and/or stabilization of such interactions between the extra-membranous protein domains is illustrated by the fact that overexpressing either the c subunit alone, or various fragments of the b subunit (including either soluble or membrane domains), or a fusion protein of the b subunit with fluorescent tag proteins, did not induce the formation of any intracellular membranes [50,51]. However, co-overexpression of b and c subunits induced the formation of internal tubules containing high amounts of both proteins [50]. The recently resolved structure of *E. coli* ATP synthase displays a dimeric association of two b subunits in the hydrosoluble part of the whole protein complex (while their intra-membranous domains are separated) [52], and this could be an indication for a possible self-assembly via the hydrophilic domain of the overexpressed b subunit. Overexpression of the *E. coli* F_0_F_1_-ATP synthase b subunit did not alter the cell membrane lipid-to-protein ratio, whereas this lipid-to-protein ratio doubled within the internal membrane tubules [50]. This change was due to a large increase (~5-fold) of CL amount at the expense of a moderate decrease of phosphatidylglycerol (PG) content, with the fatty acyl chain composition of CL remaining roughly unchanged. Fluorescent staining of the overexpressing bacteria, using the hydrophobic dyes Nile Red, FM4-64, and nonyl acridine orange (NAO), confirmed the marked increase of the lipid content, especially at the poles [51]. In particular, there was a good consistency between NAO (a probe for anionic lipids, CL, and PG) labelling and electron microscopy imaging of the intracellular membranes. A kinetic study further showed that these neo-membrane structures first developed at the cell poles, and then progressively occupied the whole cytoplasm within a 1 h period [51]. In addition, cell mutants in CL synthesis displayed a decreased formation of internal membranes, with only some multilamellar structures, but no more stacked tubules. This decrease was more important for the triple mutant of the three CL synthesis enzymes (with no CL detected, and with a concomitant dramatic increase in PG) than for a single mutant (with a CL decrease by half in the cell membrane, but an undetectable level of CL in the internal membranes) [51]. Finally, fluorescent staining allowed for flow cytometry experiments, which revealed that the bacterial population was roughly equally distributed between low and high fluorescent intensity, indicating a marked heterogeneous resistance to the protein overexpression-induced cytotoxicity.

### 3.3. Other Intracellular Membrane Structures (Poorly Defined)

The transmembrane domains of the hepatitis C virus (HCV) envelope proteins, TME1, and TME2, are reported to present a high toxicity towards their heterologous expression hosts (the strains K38 and BL21), even at very low expression levels. However, its toxicity can be decreased under various conditions [53]: expression within C41 or C43 strains (with a negligible protein production), overexpression of a glutathione S-transferase (GST)-fusion protein (the protein produced in limited amounts being present in inclusion bodies), and overexpression with the Asp-Pro sequence at the N-terminal of TME1 or TME2 (with low protein production). In contrast, protein chimeras GST-Asp-Pro-TME1 and -TME2 were produced in high amounts at 20 °C (to avoid the formation of inclusion bodies at 37 °C), with a dramatically reduced toxicity, and the expressed protein had a cytoplasmic localization. Indeed, it was found into two intracellular structures reported as “soluble micellar-like aggregates” and “proteo-lipid aggregates”, with only the latter being solubilized by a mild detergent (inefficient for the solubilization of inclusion bodies). Although these proteo-lipid structures sedimenting in the membrane fraction were not further defined, they are believed to contribute to alleviate the toxicity due to the presence of the overexpressed proteins in the cytoplasmic membrane [53]. These data show that the formation of such internal membranous structures is another way, different from inclusion body formation, to overcome the toxicity induced by membrane protein overexpression.

### 3.4. Intracellular Vesicles (“Type III” Intracellular Neo-Membranes)

A variety of membrane protein overexpressions leads to the formation of intracellular vesicles presenting more or less homogenous sizes.

#### 3.4.1. Viral Proteins

The expression in *E. coli* of the hydrophobic protein sp6.6 from the PM2 bacteriophage (not infecting *E. coli*) led to shrinkage and undulations of its cytoplasmic membrane, and to the formation of few intracellular vesicles [54]. These vesicles had similar diameter (around 50 nm) to those induced by the infection of the normal host of this bacteriophage. Thus, sp6.6 alone has a membrane morphogenesis function that should be sufficient for this infection step of the viral cycle. These observations were obtained with low expression levels of sp6.6, and it was anticipated that higher expression levels would be toxic for the *E. coli* host, probably because of cytoplasmic membrane destabilization. Similarly, the overexpression in *E. coli* of the 3A protein from the picornavirus foot-and-mouth disease virus (FMDV) induced the formation of numerous intracellular vesicles containing high amounts of the 3A protein, these vesicles being similar to those observed in the FMDV-infected cells [55].

#### 3.4.2. Lipid Metabolism Enzymes

The alkane hydroxylase AlkB (from *Pseudomonas oleovorans*) was overexpressed at a 10–15% level of total cell proteins into the W3110 *E. coli* strain while in other strains, this expression level was limited to 1.5–2%, indicating a saturating effect when located in their cytoplasmic membrane [56]. Indeed, this high overexpression level correlated to the formation of intracellular vesicles with an average diameter of 200 nm, often peripheral within the cell and with no resolved connections to the cytoplasmic membrane. These internal vesicles were found in a new, low-density membrane fraction comprising high amounts of membrane proteins, 50–70% enriched in enzymatically active AlkB that corresponded to 35–50% of all the membrane proteins. The total phospholipids content 3-fold increased while the total membrane proteins amount doubled, leading to a lipid-to-protein ratio that was moderately increased (unchanged in the cytoplasmic membrane and doubled in the internal membranes). This stimulated lipid synthesis was characterized by a doubled CL proportion, which was noticeably greater (3-fold) in the cytoplasmic membrane. A corresponding decrease of PG, with no difference between the two membranes, and a global increase of the unsaturated fatty acyl chains C16:1 and C18:1 were observed [56,57]. Addition of the phospholipid synthesis inhibitor cerulenin completely blocked the growth of the transfected and induced strain W3110 [57].

The glycosyltransferase MurG (from *E. coli*) catalyzes the conversion of lipid I to lipid II, the rate-limiting step in the biosynthesis of peptidoglycan. The overexpression of this extrinsic membrane protein in *E. coli* was associated with neo-formed intracellular membrane vesicles [58]. These vesicles were characterized by a diameter of about 50 nm, monodispersed size, marked accumulation at the cell poles, and a rather low phospholipid-to-protein ratio, but with no detectable integral membrane protein, and huge enrichment in CL (~10-fold, for a ~5-fold global increase in the cell). These observations could be correlated to the well-known CL abundance of the bacterial cell pole membranes [23], and furthermore to the specific interaction between CL and MurG, as indicated by an activation of its enzymatic activity [58].

The glycosyl transferases (from *Acholeplasma laidlawii*) MGS (monoglycosyldiacylglycerol synthase) and DGS (diglycosyldiacylglycerol synthase) are extrinsic membrane proteins whose overexpression at high levels in *E. coli* induced a massive production of intracellular membrane vesicles of various sizes (diameter from 50 to 200 nm) and shapes (sometimes appearing as “saccules”) [59]. These vesicles appeared early after protein expression induction, as soon as 15 min, and the number of vesicles progressively increased over a 22 h culture. These internal membranes formed a new, low-density membrane fraction that contained various membrane proteins, with MGS being the most abundant one. The lipid-to-protein ratio was slightly increased in both the cytoplasmic and the vesicle membranes, but the phospholipid composition was unchanged. In the case of MGS overexpression, glucosyldiacylglycerol was found at 40% of the lipids in all the membranes, since MGS could produce it from the endogenous PG. However, when overexpressing either an inactive mutant of MGS or DGS (that is unable find its substrate, monoglucosyldiacylglycerol, in *E. coli* membranes), intracellular vesicles were still formed, thus demonstrating that this formation was not dependent on the enzymatic production of an exogenous lipid [59]. In addition, fatty acid chain unsaturation moderately increased (~2-fold) while cyclopropanation showed a dramatic decrease (~6-fold), indicative of a homeoviscous adaptation that is probably required for compensating for the overproduction of the neo-membranes [60]. Regarding protein structure requirements for internal vesicle formation, mutations in the hydrophobic N-terminal segment responsible for membrane binding did not affect this capability; these observations are consistent with the fact that the 23-mer peptide encompassing this hydrophobic region did not induce any vesicle formation, thus highlighting the role of the cytoplasmic domain [59]. Indeed, deletions of 19 or 29 residues of the C-terminus region abrogated internal membranes formation (with only a partial effect for the deletion of the last nine residues) [61]. Finally, MGS was shown to specifically interact with PG and CL [62], and molecular dynamics simulations predicted that these interactions could induce a local deformation of the two lipid leaflets, a possible first step towards the formation of a global membrane curvature [61]. However, some crowding effects due to steric constraints between the protruding cytoplasmic domains could also play a role in membrane curvature generation [61].

#### 3.4.3. Caveolins

Caveolin-1 (Cav-1) and -3 are small membrane proteins (178 amino acids) required for the formation of caveolae within mammalian cells. Expression of fusion proteins of Cav-1 or -3 with maltose binding protein (MBP) in *E. coli* led to the massive formation of intracellular vesicles. These vesicles were called heterologous caveolae (h-caveolae) because of their similar size and number of caveolin molecules per vesicle compared to the caveolae, as evaluated by cryoelectron tomography [63]. The main changes in the lipid composition of h-caveolae related to cell membranes were a 2-fold decrease of CL, a 2-fold increase of lysoPE and lysoPG, and a moderate decrease in PE [63]. Remarkably, overexpression of a Cav-1 mutant, mimicking permanent phosphorylation at site S80, led to disappearance of h-caveolae, but induced the formation of intracellular membrane tubules connected to the cytoplasmic membrane [63,64]. Further analysis, using numerous Cav-1 deletion mutants to decipher the relationship between its amino acid sequence and its ability to induce the formation of h-caveolae, identified a minimal required amino acid sequence for vesicle formation. This sequence, 81–147, includes the scaffolding domain (82–101), the hydrophobic domain (102–134, intramembrane domain) and a 13-residue C-ter sequence (135–147, membrane-protected domain) required for membrane anchoring [64]. Notably, compared to the minimal caveogenic sequence, the overexpression of some protein deletion mutants allowed for the observation of intracellular neo-membranes with various morphologies, depending on the amino acid sequence of the expressed protein. Indeed, the removal of the scaffolding domain (mutant with the fused sequences 49–81 and 97–178) led to “undefined intracellular structures” made of saccules of heterogeneous sizes and shapes, whereas the deletion of the C-ter 13-residue sequence (sequence 49–134) led to extensive internal membrane tubules [64]. In addition, the overexpression of fusion proteins (with MBP) of the isoform Cav-2 or of the *Caenorhabditis elegans* homologue Ce-Cav, both known to be non-caveogenic in eukaryotic cells, did not induce h-caveolae formation. Nevertheless, these overexpressions led to significant lipid alterations with formation of intracellular neo-membranes of various morphologies, i.e., irregular tubules for Ce-Cav, and an unexpected pleiomorphic association of tubules, saccules, and vesicles, which were more or less connected to the cytoplasmic membrane, for Cav-2 [64]. These observations could indicate a relevant structural polymorphism (e.g., folding intermediates or local structural fluctuations) for Cav-2. Up to now, caveolins are the only membrane protein family that have been reported to be able to induce such different morphologies of neo-formed intracellular bacterial membranes when overexpressed. These data highlight the complex relationship between amino-acid sequence and the formation of internal membranes. They furthermore illustrate the acute relationships between protein structure and membrane morphology, in particular with a complex interplay between intramembrane and hydrosoluble domains (e.g., the intramembrane domain alone is unable to bend the cytoplasmic membrane). Further work is required to decipher this complex relationship. Nevertheless, the data on the overexpression of Cav-1 (fused with MBP) in *E. coli* showed that the protein alone is sufficient for promoting membrane budding and protrusion, and the ensuing formation of intracellular vesicles presenting a high homogeneity of size and shape. This contradicted the generally admitted requirement of various associated proteins, such as cavins and flotillins [65,66]. Conversely, the existence of caveolae-associated membrane shapes in eukaryotic cells, such as stable resting cups, grape-like clusters of vesicles or rosettes, and even elongated tubules, which was not observed in transfected bacteria, indicates the involvement in eukaryotic cells of other proteins and/or specific lipids (e.g., cholesterol).

### 3.5. Features of the Intracellular Bacterial Neo-Membranes

The combined morphological and biochemical data, acquired on intracellular vesicles and/or tubule formation in different systems, led to the following conclusions: (i) protein and lipid segregation from the cytoplasmic membrane lead to the neo-formed internal membranes; (ii) the process of membrane proliferation is likely driven by a well-suited partnership including a specific membrane protein and specific surrounding lipids; (iii) the lipid-to-protein ratio is decreased in the neo-formed internal membranes when they form tubules connected to the cytoplasmic membrane, but increased when they form vesicles/saccules/cisternae separated from the cytoplasmic membrane; (iv) CL is often reported to be involved (increased in internal membranes when measured), even if its specific role(s) is/are still misunderstood (e.g., ambiguous and complex behavior of the CL-deficient mutants); and (v) the mere initial local invagination and formation of these budding membranes seem to require different physico-chemical conditions than those required for their subsequent intracellular stabilization.

## 4. Other Cases of Membrane Stress in Different *Escherichia coli* Contexts

In addition to these various cases of overexpression of membrane proteins in *E. coli*, few other reports mention the neo-formation of intracellular membranes with various morphologies, either related to local membrane protein overconcentration or involving completely different mechanisms based on cell membrane shape alterations.

### 4.1. Tubule Formation Induced by Membrane Protein Mislocalization

Inhibition of ribosome release from the cytoplasmic membrane was obtained by disrupting either the downstream translocon (the ribosome receptor) by SecE depletion, or the SRP (signal recognition particle) machinery by Ffh (the FtsY partner) depletion [67]. Indeed, in both cases, ribosomes and FtsY largely accumulated in the bacterial membrane, while neither SecE nor Ffh influencing each other’s membrane expression. Since FtsY was, under normal conditions, only partly associated with membrane ribosomes, these data indicated that neither SecE nor Ffh was necessary for membrane binding of FtsY and ribosomes, which was consistent with a membrane targeting role of FtsY for the ribosomes. However, this accumulation of membrane-bound ribosomes and FtsY (in complexes, according to co-immunoprecipitation data) was complemented by an increase in lipid synthesis and the formation of tightly packed bundles of intracellular membrane tubules harboring high densities of ribosomes [67]. These structures were both fluorescently stained by a hydrophobic carbocyanine dye and imaged by electron microscopy, showing a good consistency.

Similar curved stacked membrane structures have been previously observed in a SecA mutant, deficient in protein export to the outer membrane; they were observed to progressively develop during a period from 0.5 to 6 h of culture [68]. This neo-membrane formation was globally accompanied by significant decreases in cellular CL content and of C18:1 fatty acid chain proportion, but remained without further characterization, in particular regarding their protein composition. Nevertheless, this neo-membrane biogenesis phenotype was reminiscent of the phenomenon previously observed in the case of the overexpression of the LamB-LacZ fusion protein, which also blocked protein export [42], thus probably also leading to a protein overcrowding of the inner membrane. This is likely similar to the case of overexpression of a membrane protein that initially accumulated in the cytoplasmic membrane.

### 4.2. Vesicles/Vacuoles Formation Induced by Bacterial Shape Alterations

Bacterial shape alterations also produce membrane stress resulting from constraints that are induced by simple geometrical considerations, which leads to membrane remodeling.

#### 4.2.1. Shape Mutants

The deletion of either one of the five “shape proteins” required for the maintenance of the rod shape of *E. coli*, MrdAB/MreBCD, led to a rod-to-sphere conversion and a decreased growth rate that could be reversed by overexpressing the division protein FtsZ. This rod-to-sphere conversion was accompanied by the appearance of intracellular vesicles [69]. These internal membrane compartments had various sizes, being often rather large (few µm), and some of them were disconnected from the cytoplasmic membrane, even if they were derived from this membrane, as indicated by their composition (the protein probe ZapA or the fluorescent lipid probe FM4-64, as well as entrapped periplasmic solutes). Since lipid synthesis rate was unchanged, this internal vesicle formation was a geometrical consequence of the rod-to-sphere conversion that led to an excess amount of membrane, due to a decreased surface-to-volume ratio, independently from FtsZ. In addition, some amounts of FtsZ, and probably other division proteins, were sequestered within these internal membranes after their fission from the cytoplasmic membrane, hence explaining the decreased division rate [69].

#### 4.2.2. L-Forms

In the specific case of the L-form of *E. coli*, cell wall-deficient cells that remain viable under normal conditions (but with various morphologies), the key division protein FtsZ level was clearly 5-fold decreased. Culture medium depletion of calcium ions induced a cell shape transformation to a sphere, followed after some hours by the formation of intracellular large, vacuole-like vesicles, which was accompanied by a reduced viability, eventually leading to cell lysis. The relationship between these observations and the calcium-dependent FtsZ polymerization required for cell division was suggested, but the mechanism of the internal membrane genesis was not further investigated, in particular regarding the possible role of FtsZ [70].

It should be noticed that, in the last two cases, at variance with data from all the above-presented cases, no local overconcentration of membrane proteins seems to play a specific role at the level of the stressed cytoplasmic membrane. The internal membrane structures formed spontaneously, probably depending on the local lipid composition, and do not have any defined morphologies. This phenomenon is somehow reminiscent of the membrane invaginations observed in pure lipid giant unilamellar vesicles under certain conditions [71,72,73].

## 5. Molecular Mechanisms

**Figure 1:**
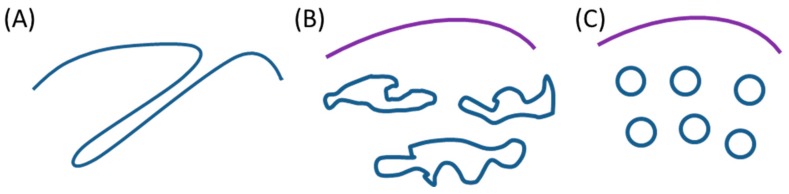
Schematic representation of three types of bacterial intracellular neo-formed membranes. (**A**) membrane tubules connected to the cytoplasmic membrane, defined as “type I”; (**B**) membrane saccules or cisternae of heterogeneous morphologies and separated from the cytoplasmic membrane, defined as “type II”; (**C**) homogenous vesicles, defined as “type III”.

The challenge of the collection of this kind of data is to decipher the underlying molecular mechanisms, especially in terms of lipid–protein interactions, involved in the membrane reorganization as a response to the membrane stress induced by a local overconcentration of a membrane protein (endogenous or exogenous, extrinsic or integral). Noticeably, the various intracellular neo-membrane structures can be classified into three typical morphologies, here called type I, II and III (Figure 1).

The various ectopic neo-membrane structures reported in Part 3 and corresponding to Figure 1 are classified in Table 1.

**Figure 2:**
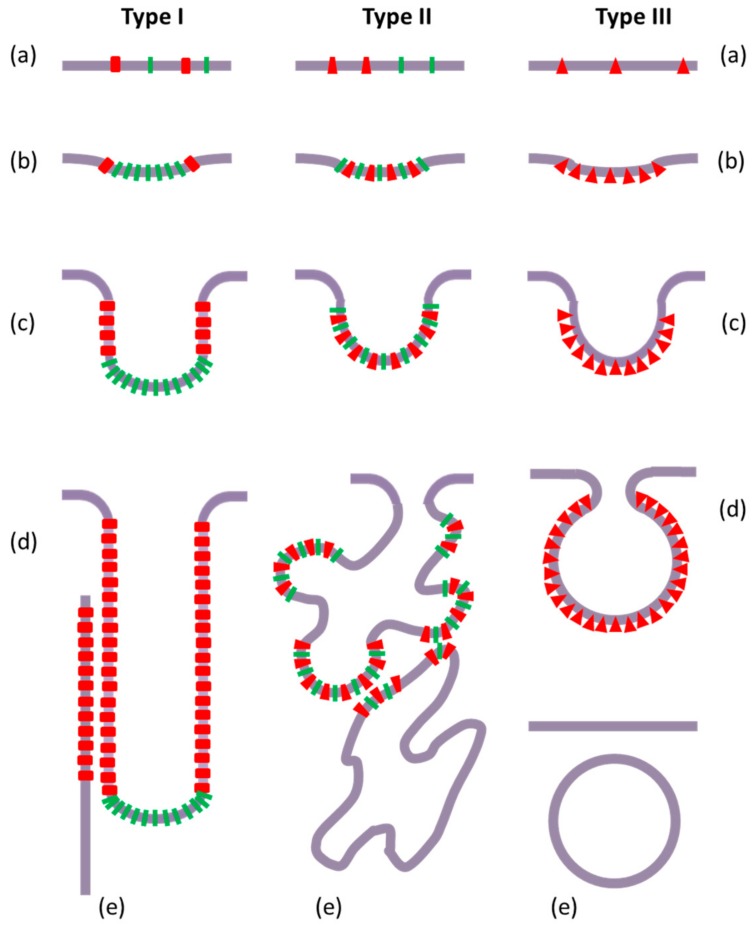
Schematic representation of formation models corresponding to the three types of bacterial intracellular neo-membranes. These three models are based on different membrane curvature-acting properties of the overexpressed membrane proteins and the surrounding lipids. Type I (connected tubules). (**a**,**b**) The initial membrane budding step (curvature initiation) results from the local assembly of curvogenic lipids (I), which is accompanied by the segregation of the overexpressed membrane protein (rectangles) at the level of the “peripheral ring” of the nascent curved membrane patch, due to the tropism of this protein for a 1D (one-dimensional) curvature. (**c**) Further local self-assembly of the overexpressed membrane protein tends to progressively elongate this peripheral membrane zone, stabilizing it in a cylindrical shape (curvature propagation). (**d**) This membrane protein self-assembly finally leads to the formation of a tubular membrane structure, still connected to the cytoplasmic membrane, with a rather low lipid-to-protein ratio (compared to the cytoplasmic membrane). (**e**) Subsequently, protein–protein interactions mediated by the hydrosoluble domains of the overexpressed membrane protein can promote stacking of such intracellular tubules (when present in high amounts). Type II (heterogeneous saccules). (**a**,**b**) The initial membrane budding step (curvature initiation) results from the local co-assembly of curvogenic lipids (I) and the 2D-curvophilic overexpressed membrane protein (trapezoids), this protein not being sufficient alone to create by itself a driving force that is able to bend the cytoplasmic membrane. (**c**) Further local co-assembly tends to quasi-spherically extend the nascent membrane patch (curvature propagation). (**d**) The more or less random clustering of the overexpressed membrane protein, associated with curvo-acting lipids (in particular with domains of different leaflet asymmetry), proceeds to promote the growth of an irregular membrane structure. (**e**) Subsequently, this membrane structure tends to eventually fission from the cytoplasmic membrane, due to energetic constraints, to finally give intracellular membrane saccules and cisternae of different sizes and shapes, with a rather high lipid-to-protein ratio (compared to the cytoplasmic membrane); stacking of these membranes can occur via interactions between the hydrosoluble domains of the overexpressed membrane protein (when present in high amounts). Type III (homogenous vesicles). (**a**,**b**) The initial membrane budding step (curvature initiation) results from local self-association of the 2D-curvogenic overexpressed membrane protein (triangles). (**c**) Further local self-assembly of the overexpressed membrane protein, associated with curvophilic lipids (not represented), leads to the formation of a hemispherical membrane (curvature propagation). (**d**) This membrane protein self-assembly progressively proceeds to build a quasi-spherical structure appending to the cytoplasmic membrane. (**e**) Subsequently, this membrane structure fissions from the cytoplasmic membrane due to energetic constraints (line tension at the level of the fission pore), to finally give intracellular spherical vesicles of homogenous size, with a rather high lipid-to-protein ratio compared to the cytoplasmic membrane (proteins not represented).

This classification is reinforced by the fact that these different morphologies follow different molecular and topological scenarios, promoting the stressed membrane budding and remodeling, depending on the respective membrane curvature-acting properties of the overexpressed proteins and the surrounding lipids (Figure 2).

### 5.1. Local Protein and Lipid Heterogeneity

Among the above-reported observations, the biochemical composition of the neo-formed membranes is most often well distinct from that of the cytoplasmic membrane, as they present a huge amount of the overexpressed protein with a large relative enrichment, along with a generally altered lipid composition (often involving CL, and sometimes unsaturated fatty acid chains). This marked difference most probably reflects a local protein and lipid heterogeneous composition at the level of the initial cytoplasmic membrane deformations and nascent internal budding membranes. Since local membrane curvature is the necessary first step for neo-formed internal membrane genesis, such lateral heterogeneity, indicates the likely involvement of “curvogenic domains”. However, these membrane domains have not yet been thoroughly investigated regarding their biophysical properties (order, defects, etc.). It should be noticed that in Gram-negative bacteria, such as *E. coli*, the cytoplasmic membrane can only bend inwards, and thanks to the mechanically protective role of the outer membrane, it should exhibit a rather low membrane tension, hence facilitating local deformations.

Typically, membrane protein–lipid domains require lateral segregation of its components within the cytoplasmic membrane, presumably by differential lateral diffusion. It is however, difficult to identify among the specific proteins and lipids recruited, those responsible for the driving force. Taking into account the well-established process of transertion, defined as “coupled transcription–translation and membrane insertion”, for bacterial membrane protein biosynthesis [14,32], a process which likely also operates for plasmid-mediated metabolism [74,75], it is tempting to propose that this local membrane protein synthesis is the first event that is responsible for the local overconcentration of the protein at the cytosolic leaflet of the cytoplasmic membrane. Indeed, for a relatively high expression level, lateral diffusion rate of the neo-proteins is likely kinetically limiting, in contrary to the lateral diffusion of endogenous lipids compared to their synthesis rate, and therefore, the local lipid composition will easily and rapidly adapt to the presence of the neo-synthesized membrane protein.

### 5.2. Local Membrane Curvature: Vesicles or Tubules

Any molecular mechanism involved in the local curvature of the cytoplasmic membrane must distinguish between vesicular, i.e., 2D-curved (“spherically shaped” with two main curvature plans), and tubular, i.e., 1D-curved (“cylindrically shaped” with one main curvature plan) deformation. Indeed, this basic feature is most probably settled from the initial membrane events, since there is no evidence reported for either the convincing coexistence of internal tubules and vesicles (except the particular, and puzzling, case of the overexpression of the fusion protein MBP-Cav-2, see Section 3.4.3 [63]), nor “pearled tubules” indicating secondary vesiculation from the preformed tubules.

In this context, it seems relevant to distinguish the molecular events that are responsible for the initiation or “nucleation”, and the propagation or “growth” of the new membrane structures. However, in both cases of vesicle and tubule formation, the first step is the deformation of the cytoplasmic membrane to a quasi-hemispheric invagination, or bud (Figure 2b). Whatever the exact relative local contributions of proteins and lipids, the theoretical descriptions of this phenomenon rely on the interplay of some physical properties of the initial membrane domain, including curvature propensity by segregated conical lipids (e.g., CL), asymmetrical distribution between the two membrane leaflets, lower bending rigidity (e.g., unsaturated acyl chains), and gain of energy resulting from a decreased line tension (indicating domain contribution) [76,77,78], along with specific or non-specific protein interactions with this membrane patch [79,80,81,82]. In few cases, such as the extrinsic proteins containing F-BAR (Bin/Amphiphysin/Rvs) domains that are sufficient for membrane curvature initiation, there could be no requirement or involvement of curvo-active lipids [83]. As a matter of fact, the neo-synthesized membrane proteins can induce, or contribute to, a local membrane curvature by five main mechanisms: (i) the oligomerization creating a “coat” that interacts with the lipids and enforces them a specific shape; (ii) the hydrophobic wedge effect, as a consequence of the insertion of a conic transmembrane domain of an integral membrane protein, or of a semi-integral (i.e., inserted in only one leaflet, as proposed for caveolins) membrane protein; (iii) the (more or less deep) adsorption at the membrane interface of an helix (typically amphipathic) or an hydrophobic anchoring domain (as proposed for MGS) from an extrinsic membrane protein; (iv) the crowding effect, inherently entropic, due to the steric constraints of a high concentration of hydrosoluble domains of membrane proteins, impeding their free diffusion and creating a local pressure deforming the membrane, which becomes convex to minimize protein collisions [84,85,86,87]; and (v) the protein functional role if related to lipid metabolism or transfer.

For the next step, i.e., the growth of the new membrane structures, the initial membrane deformation (“inward budding”) will be submitted to two possible evolutions, depending on the propensity of the locally accumulated membrane protein to associate to a curved membrane (Figure 2c): either a spherical 2D-curvature (type II and type III neo-membranes) in the center of the budding patch, or a cylindrical 1D-curvature (type I neo-membranes) at the periphery of this protruding membrane patch, around the “neck”, i.e., the inflection point of the bud, where the double curvature progressively reverses [81], (as recently observed for cholera toxin B subunit [88]). Clearly, the preference of the membrane protein depends on the geometrical features of its structure, especially for whether it has a pseudo-symmetry axis that is perpendicular to the membrane plan (leading to a double local curvature), or in contrast, whether it displays a markedly elongated shape within the membrane plan (defining the axis of the single curvature). At this stage, it is important to note that a membrane protein would efficiently induce and stabilize a membrane curvature only if it is able to mediate a collective behavior, since an isolated protein could only very locally perturb the membrane, but without inducing a sustained and stable deformation resistance to thermal fluctuations (as deduced from simple energetic considerations) [80,82,89]. However, such a membrane protein clustering can be provided by either specific self-assembly or indirectly mediated by the membrane, such as lipid shell fusion, specific lipid repulsion, minimization of hydrophobic mismatch, or membrane undulation inhibition [90]. Anyway, the monomer, as well as the self-assembly, could display the structural features of the curvature-active membrane protein.

Membrane proteins which accumulate at the budding domain periphery progressively build a “ring” making use of the local 1D-curvature, hence expanding and eventually forming a tubular neo-membrane structure (with an increased lipid-to-protein ratio), here so-called type I. These cylinder-shaping membrane proteins, at least the extrinsic proteins, should have a “curve-sensor” domain with an elongated geometry, either an elongated global shape (typically “banana-shaped” [91]) perpendicular to the cylinder axis, or a hydrophobic helix inserted at the membrane interface parallel to the cylinder axis [92]. 1D-curvature genesis requires the formation of a protein array defining a preferred direction [89], as actually observed for Frd and PlsB [45,46]. In particular, such 1D-curvature can be a promoting factor for rod-like membrane proteins aggregation under certain conditions of attractive interactions and high concentration [93]. The cylindrical shape and diameter are then obtained by adapting the protein-promoted 1D-curvature with the physical parameters of the membrane (bending rigidity and curvature-propensity of the lipids), in particular, tilting with respect to the axis and/or adjusting the helix pitch of the protein array around the formed membrane tubule [93].

Alternatively, the case of sphere-shaping membrane proteins is especially represented by coat-forming proteins (e.g., clathrin and caveolin) whose oligomers structures are specifically designed to build spheroid objects (with a decreased lipid-to-protein ratio), here so-called type III neo-membranes. A spherical deformation, eventually leading to vesiculation, can also be promoted by protein crowding, which inherently presents an isotropic symmetry with respect to the membrane plan (without advantaged direction) [94,95], as well as by the widely described wedge effect, which does not necessarily involve oligomerization, and can lead to the formation of the here so-called type II neo-membranes (see below).

Obviously, in all these membrane protein-mediated curvature-active steps, the endogenous membrane lipids are directly or indirectly involved, contributing to the stabilization of the “curvogenic domain”. Schematically, the curvature-prone lipids are those presenting a conic shape, with either a small headgroup (such as CL) contributing to a concave leaflet, or a large headgroup (such as lysolipids) contributing to a convex leaflet [82]. Consequently, they can be segregated in one specific membrane leaflet within the curved membrane domains (even in the absence of specific interaction with the accumulated membrane protein), and it has actually been shown that this “sorting” is more efficient when performed collectively [76,96]. Since CL is a rather abundant phospholipid in *E. coli* cytoplasmic membrane and it is involved in membrane lipid domains [23], especially in regions of high curvature (poles and division septum) [19,28], it is not surprising that it is very often found to be enriched in the internal neo-membranes observed in *E. coli*. However, the use of CL synthesis deficient mutants indicates a rather ambivalent role of CL, since perhaps this lipid is more closely involved in tubule stabilization rather than in the initial formation, such as in the cases of Frd and the b subunit of the F-ATP synthase [45,51]. Furthermore, considering the initiation or nucleation step of inward budding formation, it has already been proposed and demonstrated in some cases that the transertion structure in the cytoplasmic membrane should be a specific domain involved in lipid (including CL) segregation due to preferential interactions with the neo-synthesized membrane protein, especially if expressed at high levels [14,32]. In addition, polyunsaturated fatty acid chains are often found in tubular and vesicular structures, in line with their reported role in model membrane deformation [97]. Two remaining points have still to be clarified: the additional role of preexisting cytoplasmic membrane domains, such as those characterized by the presence of the flotillin homologue, FloT, and the likely involvement of endogenous lipid translocases that are responsible for transversal asymmetry, and hence the local curvature, of the cytoplasmic membrane.

In summary, bacterial internal membrane formation is rather structurally demanding, since the various underlying molecular scenarios always rely on stringent conditions regarding the structure of the proteins, either as a monomer or within oligomers or clusters, combined with specific interactions with the surrounding lipids. This could explain why such internal neo-membranes are so rarely observed for few membrane proteins. As an illustration, two systematic proteomic and genomic investigations of the *E. coli* response to overexpression of various transmembrane domain-containing proteins (three green fluorescent protein (GFP)-tagged membrane proteins, YidC, YedZ, and LepI, six polytopic membrane transporters, GlpT and MsbA, and four homologues and mutants) reported only the accumulation of these proteins in “cytoplasmic aggregates” and inclusion bodies, but showed no ectopic intracellular membranes [98,99]. But such a point obviously requires further systematic investigations.

### 5.3. Membrane Structures More or Less Dispersed in Size, Shape, and Order

In fact, for both vesicular and tubular structures, the experimental observations showed that the neo-formed internal membranes present either mono- or polydispersed sizes and shapes (Figure 2d). The observation of a defined size for either a spherical or cylindrical membrane structure (“calibrated vesicles and tubules”, the type III and type I neo-membranes, respectively) indicates a strong geometrical constraint, which results from the polymerization of the membrane protein or its specific assembly, and highlights the strength of such protein-protein interactions. On the contrary, variations in the sizes and shapes of these structures (“saccules” and “tubular invaginations”, the type II neo-membranes) can rather be the result of a membrane-mediated protein sorting mechanism that is promoted by the local curvature, and based on individual behavior (in contrast to lipid molecules) [96]. Thus, these structures are more likely involve the interplay of various non-specific lipid–protein and lipid–lipid interactions. Such a specific morphology is probably due to the feature of the locally accumulated membrane protein, which can be either “curvogenic”, i.e., endowed with a driving force, thanks to self-association capabilities, sufficient for mediating an active segregation deforming the membrane (e.g., cholera toxin B subunit and caveolin-1), or “curvophilic”, i.e., only endowed with the capacity for passive segregation (or sorting) into an already curved membrane, as previously reported for ArfGAP1 and the amphipathic lipid-packing sensor (ALPS) motif-harboring membrane proteins [100,101]. This difference should be linked to the energy involved in protein–protein and protein–lipid interactions compared to the energy that is required for membrane deformation [82].

In addition, supra-membranous orders are sometimes observed for neo-formed tubules, appearing as homogeneous stacks in the bacterial cytoplasm. These typical intracellular structures likely formed subsequent to the initial tubule generation, and probably result from specific interactions between the hydrosoluble domains of the locally over-concentrated membrane proteins. Obviously, such rare events give good opportunities for structural studies, thanks to these quasi-crystal arrangements.

### 5.4. Membrane Fission or Continuity

The current models for membrane fission, based on both theoretical and experimental data on eukaryotic cells, rely on the essential involvement of protein machinery (typically dynamin and ESCRT-III complex) to perform membrane fission, although the role of lipids in the intermediate structure is still insufficiently characterized [102]. In *E. coli*, no protein machinery contributing to the release of the neo-formed vesicles or tubules from the cytoplasmic membrane has been described. In addition, in the case where the locally highly concentrated membrane protein (devoid of any fission function) is curvature-active (curvogenic or curvophilic), this protein cannot be present simultaneously in both the convex budding region of the stressed membrane and in its neck (inversed curvature) where the fission process takes place. Thus, in agreement with the experimental observations reported in this review, we propose to consider the two following exclusive cases (Figure 2): (i) the protein of interest participates to the expansion of the initially budding membrane (type II and III neo-membranes), or (ii) it is confined to its peripheral region where it ensures a local membrane stabilization counteracting the narrowing of a “fission pore” (type I neo-membranes). In the first case, the progressive expansion of the budding membrane will irreversibly and ineluctably lead to a destabilization of the bud-neck, which becomes more and more curved, and the stressed bilayer tends to minimize its bending elastic energy, together with line tension in the case of local lipid segregation and eventually (above a certain threshold) fuses to give a separated closed vesicle [103,104,105]. The absence of any “specific fission protein” in the bud-neck is reminiscent of the observation of microvesicle fission from a pure lipid giant unilamellar vesicles that have been submitted to various physical (thermal or osmotic) stresses [72,73,106]. Thus, it is very likely that the protein–lipid domain driving the budding membrane formation indirectly generates the line tension. Obviously, it is difficult to know the exact molecular scenario involved in the membrane transition state, in particular, whether it implicates an hemifusion structure (hence avoiding any leak of the periplasmic medium) or not. One must recall here that the periplasmic leaflet of the cytoplasmic bacterial membrane is associated with peptidoglycans that can play a role in membrane interaction and/or membrane fusion. In the second case, the progressive accumulation of the membrane protein in the bud-neck will lead to the formation of a long growing stalk with a single curvature (i.e., cylindrical in shape), to finally produce a typical tubular membrane structure that is connected to the cytoplasmic membrane (tubular invaginations). Thus, the two apparently independent morphological features, vesicles/saccules vs. tubules, and separated vs. connected, consist currently of only the two exclusive cases, detached vesicles vs. connected tubules, depending on the molecular mechanism that is used for propagating the initial membrane deformation.

## 6. Some Remaining Questions

Beyond shedding some light on the reported data, such a literature review also raises some remaining questions, in order to stimulate further studies, both in the basic and applied fields.

### 6.1. Ectopic Neo-Formed Intracellular Membranes in Other Bacteria

As far as we know, the description of ectopic intracellular membrane generation in other bacteria than *E. coli* has not been reported yet. The Gram-positive bacteria *Lactococcus lactis* is well known to allow for high-level production of exogenous membrane proteins without forming inclusion bodies [107]. Therefore, the formation of internal neo-membranes within these bacteria could be a convenient means for resisting against membrane stress and cell toxicity that is induced by the accumulation of the neo-synthesized proteins, but this has not been addressed yet. This clearly requires further morphological and biochemical investigations in *L. lactis* and other bacteria.

### 6.2. Toxicity Mechanisms

A rather common observation when overexpressing membrane proteins in *E. coli* is a marked toxicity for the host cells, varying from a more or less stringent decrease of the growth rate to complete lethality, which depends on the expression level. However, this bacterial cytotoxicity depends on the nature of the overexpressed membrane protein, but this relationship is still not understood and requires a case-by-case analysis [98]. Indeed, this toxicity could be associated with a specific function of the membrane protein (e.g., lipid metabolism), and/or the membrane protein structure, including the induction of deleterious membrane defects, or a global non-specific perturbation of the cell metabolism that is submitted to a biochemical highjacking, allowing for a high-level lipid synthesis. Moreover, saturation of the Sec translocon capacity, combined with an altered energy metabolism, has been pointed in some cases [99]. It could be valuable to find selected strains that can resist such toxicity mechanisms, as is performed for the b subunit of F_0_F_1_-ATP synthase [50], and the HCV TME1 and TME2 envelope proteins [53], since this could give information on the underlying processes that are related to the overexpression of a considered protein. It has been reported that the unusual potency for expressing high levels of membrane proteins of the *E. coli* strains C41 and C43 was related to a high activity of the T7 RNA polymerase [108], but this observation does not address the relationship with intracellular neo-membrane proliferation. Anyway, this phenomenon appears as an efficient way for the bacteria to become resistant against the accumulation of too high an amount of an overexpressed membrane protein, finally providing another protection pathway besides the widely reported inclusion body formation. A key issue is then to determine the criteria leading to the selection of one of these pathways. It is perhaps noteworthy that PlsB overexpression in intracellular tubules was accompanied by the activation of the heat shock proteins GroEL and DnaK, while in a heat shock protein mutant strain, this overexpression induced inclusion body formation [47]. Conversely, overexpression of DnaK/J (and of GroEL/ES, but with a more limited effect) associated with the membrane transporter CorA reduced inclusion body formation, but without intracellular neo-membrane generation [109].

### 6.3. Neo and Extra Lipid Synthesis

The high-level synthesis of lipids linked to the formation of internal neo-membranes is another puzzling point. Among the unanswered questions are the following ones: What is the initial sensor that triggers this neo-synthesis? Is it essentially non-specific, or does the nature of these extra lipids depends on the overexpressed membrane protein, or on other criteria, such as neo-membrane morphology? Do these extra lipids contribute to the initial local membrane curvogenesis, or do they simply accompany the curvogenic effect of the overexpressed membrane protein? A possible answer to the first question could be a regulation of PlsB (the lysophosphatidic acid synthase) mediated by the intermediate metabolite guanosine tetraphosphate [110]. However, PlsB overexpression induced intracellular tubule formation that was rather comparable to that observed with the overexpression of Frd, an enzyme without any connection with lipid biosynthesis metabolism.

### 6.4. Protein–Protein Interactions

In the case of a local accumulation of an overexpressed membrane protein, these proteins likely interact specifically or not. In addition, as mentioned above, protein–protein interactions are required for membrane curvogenesis. These interactions depend on the various structural characteristics of the protein, and it is worth being specifically addressed for each case. We will distinguish hydrophobic interactions within the membrane involving the surrounding lipids, and hydrophilic interactions in the cytoplasmic medium, which implicate either extrinsic membrane proteins or the cytoplasmic domains of integral membrane proteins. Such interactions could theoretically be predicted by molecular simulation (protein–protein docking) if the protein structure is known with a sufficient resolution. If the structure is unknown, alternatively, these ectopic bacterial membranes that are highly enriched in a given protein can be prone for structural studies, such as 2D crystal diffraction using stacked tubules, or solid state NMR using neo-formed isolated vesicles.

For membrane protein biosynthesis, the most sensitive step is the lipid insertion of the nascent hydrophobic polypeptide, before its further elongation and final correct folding. Knowledge about the SRP pathway and the translocon complex SecA–SecYEG leads to the hypothesis that the protein YidC could play a dual role of an insertase of the new transmembrane segments, and of chaperone for the growing membrane protein, especially for a polytopic and/or oligomerized membrane protein [111,112]. Specifically, YidC has been reported to be involved in the biosynthesis of fumarate reductase and of the hydrophobic subunits of F_1_F_0_-ATP synthase. Therefore, its role (and even requirement) in the case of overexpression of a membrane protein leading to a proliferation of intracellular membranes neo-formed from a hyperactive transertion process, is questioned. Does YidC have a stabilizing action on the new membrane protein? Is it specific to this protein? Does it contribute to the initial curvature of the budding membrane patch?

Another related question addresses the co-overexpression of two membrane proteins, with one of them being able to induce neo-formed intracellular membranes, as reported for b and c subunits of the F_1_F_0_-ATP synthase [50], and which is proposed for other membrane proteins in association with the b subunit [113] or with Cav-1 [114]. The questions about the fate of the second membrane protein and the ways to predict it are intriguing. In particular, does a “co-transertion” process take place? Are the two membrane proteins present within the neo-formed ectopic internal membranes? Such a possibility could depend on either a passive segregation into the new extra membrane reservoir (“tank effect”) or a specific association (“scaffold effect”) of the two proteins. This is actually an important issue in the applied perspective of membrane protein overproduction.

### 6.5. Stress-Induced Morphological Membrane Changes

Besides the various stresses to which a membrane can be submitted, such as mechanical (shearing), electrical (electroporation), chemical (lipid peroxidation), and chemico-physical (surfactant insertion) ones, the presence of high amounts of a given membrane protein can also induce a non-physiological response, although still possibly compatible with cell survival. This response consists of the local curvature and further remodeling of newly formed membrane patches that are specifically enriched in the considered membrane protein. This has been observed in *E. coli* for a limited set of proteins that are generally not involved in membrane morphology and trafficking. Alternatively, when the protein is suspected to be involved in such mechanisms (e.g., viral proteins, caveolin), the observation of intracellular neo-membranes demonstrates that this protein is sufficient alone for inducing such membrane deformation. However, beyond this aspect of membrane stress, it can be anticipated that these curvature-acting (curvogenic or curvophilic) membrane proteins, when present at low concentrations, should inherently display some kinds of local membrane perturbing effects, which could be part of its physiological function (e.g., viral proteins, lipid metabolism enzymes, etc.). Thus, such observations of neo-formation of ectopic intracellular membranes induced by local membrane protein accumulation should not only be considered as objects of curiosity, but also as a means for revealing the underlying molecular mechanisms involving lipid–protein interactions. These ectopic intracellular membranes are thus worth being further addressed in the future.

## Figures and Tables

**Table 1:** Overview of the different membrane proteins whose overexpression in *E. coli* induces the formation of ectopic intracellular neo-membranes. These proteins are classified according to the three morphologically distinct types of neo-membranes, as shown in Figure 1.

Type	Overexpressed Membrane Protein	References
I	Fumarate reductase complex (FrdABCD)	[40,45]
	sn-Glycerol-3-phosphate acyl transferase (PlsB)	[41,46,47]
	LamB-LacZ fusion protein	[42]
	Mannitol permease (MtlA)	[43]
	Chemotaxis receptor (Tsr)	[44]
	Pseudo-phosphorylated mutant S80/Cav-1 ^1,a^	[63,64]
	Truncated Cav-1(49–134) ^1,b^	[64]
	Caveolin-2 (Cav-2) ^1^	[64]
II	Lipid A disaccharide synthase (LpxB)	[48]
	Lipid A disaccharide synthase (LpxB) ^2^	[48]
	F_0_F_1_-ATP synthase	[49]
	F_0_F_1_-ATP synthase b subunit	[50,51]
	Truncated Cav-1(49–81/97–178) ^1,c^	[64]
	Nematode caveolin (Ce-Cav) ^1^	[64]
	Caveolin-2 (Cav-2) ^1^	[64]
III	sp6.6 protein of PM2 bacteriophage	[54]
	3A protein of foot-and-mouth disease virus (FMDV)	[55]
	Alkane hydroxylase (AlkB) ^3^	[56,57]
	Glycosyl transferase (MurG)	[58]
	Monoglycosyldiacylglycerol synthase (MGS) ^4^	[59,60,61,62]
	Diglycosyldiacylglycerol synthase (DGS) ^4^	[59]
	Caveolin-1 (Cav-1) ^1^	[63,64]
	Truncated Cav-1(81–147) ^1,d^	[64]
	Caveolin-2 (Cav-2) ^1^	[64]

^1^ All caveolin homologues and mutants originate from mammals, except the nematode homologue Ce-Cav that is from *Caenorhabditis elegans*; all proteins were overexpressed as fusion proteins with the maltose-binding protein (MBP). ^a^ Point mutant mimicking permanent phosphorylation. ^b^ Truncated protein keeping the sequence 49–134. ^c^ Truncated protein keeping the fused sequences 49–81 and 97–178. ^d^ Truncated protein keeping the sequence 81–147. All other proteins originate from *E. coli* except otherwise indicated: ^2^ from *Haemophilus influenzae*, ^3^ from *Pseudomonas oleovorans*, ^4^ from *Acholeplasma laidlawii*.
